# A community-based participatory research methodology to address, redress, and reassess disparities in respiratory health among First Nations

**DOI:** 10.1186/s13104-015-1137-5

**Published:** 2015-05-16

**Authors:** Punam Pahwa, Sylvia Abonyi, Chandima Karunanayake, Donna C Rennie, Bonnie Janzen, Shelley Kirychuk, Joshua A Lawson, Tarun Katapally, Kathleen McMullin, Jeremy Seeseequasis, Arnold Naytowhow, Louise Hagel, Roland F Dyck, Mark Fenton, Ambikaipakan Senthilselvan, Vivian Ramsden, Malcolm King, Niels Koehncke, Greg Marchildon, Lesley McBain, Thomas Smith-Windsor, Janet Smylie, Jo-Ann Episkenew, James A Dosman

**Affiliations:** Canadian Centre for Health and Safety in Agriculture, University of Saskatchewan, 104, Clinic Place, Saskatoon, SK S7N 2Z4 Canada; Department of Community Health and Epidemiology, University of Saskatchewan, Health Science Building, 104, Clinic Place, Saskatoon, SK S7N 5E5 Canada; Community A, Saskatchewan, Canada; Community B, Saskatchewan, Canada; Department of Medicine, University of Saskatchewan, Saskatchewan, Saskatoon Canada; Simon Fraser University, Burnaby, British Colombia Canada; Department of Academic Family Medicine, University of Saskatchewan, Saskatchewan, Saskatoon Canada; Johnson-Shoyama School of Public Policy, University of Regina, Saskatchewan, Regina Canada; First Nations University of Canada, Saskatchewan, Regina Canada; University of Toronto, Toronto, ON Canada; Indigenous Peoples’ Health Research Centre, University of Regina, Saskatchewan, Regina Canada

## Abstract

**Background:**

To date, determinants of respiratory health in First Nations people living on reserves and means of addressing and redressing those determinants have not been well established. Hence the Saskatchewan First Nations Lung Health Project (FNLHP) is a new prospective cohort study of aboriginal people being conducted in two First Nations reserves to evaluate potential health determinants associated with respiratory outcomes. Using the population health framework (PHF) of Health Canada, instruments designed with the communities, joint ownership of data, and based on the 4-phase concept of the First Nations Regional Longitudinal Health Survey, the project aims to evaluate *individual* factors, *contextual factors*, and principal covariates on respiratory outcomes. The objective of this report is to clearly describe the methodology of (i) the baseline survey that consists of two components, an interviewer-administered questionnaire and clinical assessment; and (ii) potential intervention programs; and present descriptive results of the baseline data of longitudinal FNLHP.

**Methods:**

The study is being conducted over 5 years (2012–2017) in two phases, baseline and longitudinal. Baseline survey has been completed and consisted of (i) an interviewer-administered questionnaire-based evaluation of individual and contextual factors of importance to respiratory health (with special focus on chronic bronchitis, chronic obstructive pulmonary disease, asthma and obstructive sleep apnea), and (ii) clinical lung function and allergy tests with the consent of study participants. The address-redress phase consists of potential intervention programs and is currently being rolled out to address-at community level (via green light program and environmental study), and redress-at policy level (via obesity reduction and improved diagnosis and treatment of obstructive sleep apnea) the issues that have been identified by the baseline data.

**Results:**

Interviewer-administered surveys were conducted in 2012–2013 and collected data on 874 individuals living in 406 households from two reserve communities located in Saskatchewan, Canada. Four hundred and forty six (51%) females and 428 (49%) males participated in the FNLHP.

**Conclusions:**

The information from this project will assist in addressing and redressing many of the issues involved including the provision of adequate housing, health lifestyle practices, and in planning for health service delivery.

**Electronic supplementary material:**

The online version of this article (doi:10.1186/s13104-015-1137-5) contains supplementary material, which is available to authorized users.

## Background

In Canada, Aboriginal Peoples’ (First Nations, Metis, and Inuit) [1] respiratory health inequalities pose exceptional challenges that are both complex and complicated. For instance, Aboriginal populations experience greater smoking levels [2], increased indoor air pollutants due to inadequate housing [2-4], and higher rates of obesity [2-5], which in itself is linked to several respiratory health outcomes [2-8]. These challenges could be attributed to inequities such as Aboriginal Peoples’ socioeconomic disadvantages [9], which are driven by historical injustices, including colonization [10] and residential school enrollment [11,12]. There is an intergenerational link between attendance at a residential school, smoking and chronic bronchitis [12,13]. Poor housing conditions such as housing in need of major repairs; [14] dampness and mold; [15-18] adverse indoor air quality due to overcrowding; and both active and passive cigarette smoke [3,8] lead to respiratory diseases [19-23]. The purpose of this manuscript is to explain the methodology of the First Nations Lung Health project that aims to test the hypothesis that living conditions in many First Nations communities characterized by personal, social and physical environments are associated with adverse respiratory outcomes.

To understand the magnitude of these respiratory health inequalities, it is important to contextualize this issue in terms of the Canadian population. According to the 2011 National Household Survey (NHS), the Indigenous Peoples’ Population was 1.4 million, representing 4.3% of the total Canadian population [24]. Within Canada, Manitoba (16.7%) and Saskatchewan (15.6%) are the two provinces with highest proportion of self-identified Indigenous people and it is projected that Saskatchewan will account for 20% in 2015, 28% in 2035, and 33% in 2045 of the total Indigenous population in Canada [25]. More specifically, Canada's Indigenous population is growing faster than the general population [24]. The bulk of this increase is represented by the younger Indigenous population, with the median age of the total Aboriginal population being 27.7 years, which is 13 years lower than the median age of non-Indigenous population [24]. These statistics highlight two critical implications. First, the faster growth of the Indigenous population highlights the widening health inequalities which would be unsustainable with current trends in Canadian population growth. Second, with poor household conditions increasing the risk of chronic respiratory illnesses in children as well, the considerably faster increase in younger Indigenous population puts a large proportion of them again at risk of the intergenerational transmission of health inequalities.

In Saskatchewan, an estimated 52% of First Nations people live on reserves [14], and these reserves are categorized as non-isolated (73%), semi-isolated (15%), isolated (9%) and remote isolated (2%) [2]. Moreover, with access to health care in terms of geography being an important determinant of health [26-28] and with “place” being an important population health variable [29], the respiratory health risks faced by Indigenous Peoples are further accentuated in rural Indigenous populations due to their geographic and economic isolation.

Irrespective of these poor health outcomes, First Nations peoples’ survival under exceptionally difficult conditions is evidence of their resilience. Tackling these disparate health outcomes using interventions created in collaboration with First Nations communities using community-based participatory research (CBPR) methodologies would focus on this resilience. Furthermore, this approach would provide evidence that participatory methodology is ideal to influence change and build capacity in Aboriginal communities. Thus, a CBPR initiative involving two First Nations communities in Saskatchewan is being implemented in multiple stages to foster participatory action research. To date, determinants of respiratory health in First Nations people living on reserves and means of addressing and redressing those determinants have not been well established, and form the core of the activities of the Canadian Institute of Health (CIHR) funded study entitled ‘Assess, Redress, Re-assess: Addressing Disparities in Respiratory Health Among First Nations People’ (henceforth known as the First Nations Lung Health Project [FNLHP]). Based on Health Canada’s Population Health Framework [30-32], the FNLHP, by understanding how *individual* and *contextual* factors influence adverse respiratory outcomes, aims to implement appropriate community-level (address) and policy-level (redress) interventions in two on-reserve First Nations communities in Saskatchewan (henceforth known as Community A and Community B). The objective of this report is to clearly describe the methodology for (i) the adult component of the baseline survey that consists of two components, an interviewer-administered questionnaire and clinical assessment; and (ii) the potential intervention programs; and present descriptive results of baseline data of the longitudinal FNLHP.

### Saskatchewan First Nations people living on reserves

According to the 2011 NHS, the population of Aboriginals in Canada comprised of First Nations (851,560), 413,380 Métis (413,380), and Inuit (59,115) [24]. In Saskatchewan: First Nations (103,210), Métis (52,450) and Inuit (290), in total representing 15% of the provincial population. In Saskatchewan, an estimated 52% of First Nations people live on reserves [25].

## Methods

### Phase 1: Vision and relationships leading to problem identification

**Figure 1:**
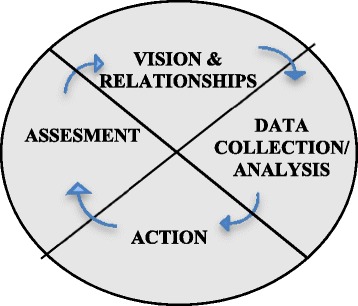
Four-phase approach of the First Nations regional longitudinal health survey.

The project was conceptualized and developed through two years of “Vision and Relationships” dialogue with the participating communities. Ten consultation sessions were held with community leaders, health workers and community members (Figure 1). Four exposure − outcome dyads were identified by the communities as key factors driving respiratory health inequalities: i) the quality of housing and mold within the houses − asthma, especially in children; ii) smoking in general, and smoking in the homes resulting in environmental tobacco smoke (ETS) − chronic obstructive pulmonary disease (COPD); iii) overcrowding and infections − bronchitis; iv) body weight − obstructive sleep apnea (OSA). A Decision Makers Council consisting of Band Councilors, elders and youth was then formed to oversee the FNLHP. Table 1 is the logic model for the FNLHP which enumerates the four phases of the study, including how the four exposure-outcome dyads were assessed and will be addressed and redressed. An agreement was signed that addressed issues on co-ownership of data between researchers and communities and how confidentiality and privacy would be respected.

**Table 1:** **FNLHP logic model**

**Issue**	**Assess**	**Address**	**Redress**	**Reassess**
**(Identified problems)**	**(Baseline)**	**(Community-level)**	**(Policy-level)**	**(Outcome measures)**
1. Housing	-Environmental measures	- House keeping	-Household mold remediation	-Reduction in wheezing in children
- dampness, mold, endotoxin	-Asthma in children	- Managing asthma	- Housing policy	- Reduction in smoking in houses with children
-Environmental tobacco smoke		-“Outdoor living room” (not smoking)		
- Wood/oil heating				
2. Smoking	-Symptoms	- “Breath of Fresh Air Campaign”	- Support for culturally appropriate smoking cessation	- Reduction in smoking in graduating grade 12
	-COPD	- Management of COPD		- Improvement in lung function
	-Lung function			
3. Infections	-Bronchitis	- Immunization	- Housing policy (crowding)	- Reduction in flu cases and respiratory infections
- Over crowding		- Flu vaccine		
		- Prompt treatment		
4. Body weight	-Sleep Apnea	- Identify cases	- Access to healthy/nutritive food	-All diagnosed cases of sleep apnea treated
		- Community sports	- Equipment for treating sleep apnea	- Reduction in average weight
		- Combined initiative with diabetes programs		

### Phase 2: Baseline assessment ─ data collection and analysis

Phase 2 corresponds to the baseline survey, which has been completed. In each of the two participating First Nations communities the baseline assessment consisted of two stages. The first stage involved personal invitations via door-to-door canvassing to make people aware of the baseline survey and distribute brochures explaining the need and purpose of the study. The second stage consisted of the in-person administration of questionnaires and conducting clinical assessments. Before conducting the survey, a Certificate of Approval was obtained from the University of Saskatchewan’s Biomedical Research Ethics Board. Moreover, before implementing the second stage of this phase, informed written consent from all participants was obtained.

### Questionnaire development and administration

**Figure 2:**
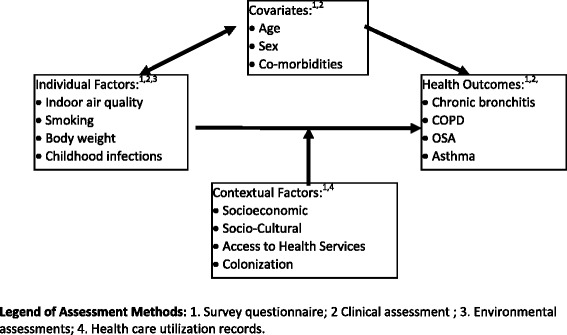
Population health framework.

Two questionnaires were administered to adults in the second stage of the baseline survey. These included a household questionnaire and an individual questionnaire. In developing the baseline assessment questionnaires (household and individual), PHF’s framework was taken into account by including questions that capture both individual and contextual factors (see Figure 2). However, before developing the questionnaires, feedback was obtained from community advisors (elders and health director) of both participating reserves. Community advisors also provided input about the best approaches for contacting participants; as well as collecting questionnaire and clinical assessment data from participants. Finally, a pilot study was then conducted to optimize the content and administration of the baseline questionnaires. Based on the pilot project responses, several survey questions were modified in the questionnaires that were finally used for the FNLHP baseline survey conducted between 2012 and 2013. Trained research assistants from each community undertook to personally invite every community resident (18 years and older) via door-to-door canvassing to visit the Health Centre in the community to complete the interviewer-administered questionnaires and to participate in clinical assessments. An identification number was assigned to each household using the community map. During their visit to the Health Centre, study participants were asked to identify their respective households on the map. An interviewer-administered survey consisted of asking a key informant from each household to provide household level information (household questionnaire). In addition, adults (18 years and older) who visited the Health Centre were also asked to complete the individual section of the questionnaire and invited to undergo pulmonary function and allergy skin prick testing.

The questionnaires were primarily designed to obtain information on individual and contextual determinants that could influence respiratory health. However, questions were also included to evaluate general respiratory health, including any history of other health conditions.

### Baseline pulmonary function and allergy skin tests

All baseline clinical tests were conducted by trained health professionals. *Sensormedics* (Anaheim, CA) dry rolling seal spirometers [33-35] were used to obtain measures of forced vital capacity (FVC), forced expired volume in one second (FEV_1_), FEV_1_/FVC ratio × 100, and maximum mid-expiratory flow rate (FEF_25–75_). Measurements were taken according to the standards of the American Thoracic Society [36]. Persons who demonstrated below normal tests were referred to their family physician for follow-up.

Allergy skin tests were conducted using the skin prick method with a panel of six non-food allergens: cat dander, local grasses, *aspergillus sp*, alternaria, house dust mite, cladosporium, along with control saline and histamine as the positive control. Standardized allergen extracts were used as recommended by the Academy of Allergy, Asthma, and Immunology [37]. Also, the allergy skin test procedure was performed according to the recommended protocol of practice parameters for allergy diagnostic testing. Subjects were considered positive for atopy if one or more skin prick tests resulted in a raised wheal that is greater than 3 mm compared to the saline control.

### Variables of interest

#### Outcomes

Outcomes of interest were from the survey as well as the clinical visit. The key clinical outcomes (self-reported doctor diagnosed) of the FNLHP from the survey are asthma, COPD, chronic bronchitis and OSA. The key outcomes from the clinical testing were the lung function measurements (FVC, FEV_1_/FEV1/FVC, and FEF_25–75_) obtained via spirometry to ascertain respiratory health.

#### Contextual factors

The primary contextual factors associated with respiratory health outcomes are crowding (based on number of people who usually live in the household and number of bedrooms); socioeconomic status (assessed using total household income, household income adequacy, and perceived financial strain); socio-cultural factors (family social support, community social support, colonization [see Additional file 1]); access to health care services; and indoor air quality (assessed by response to questions about the quality of house – in need of major/minor repairs, water damage and dampness, mildew, and presence of proper ventilation, and indoor smoking). More importantly, in collaboration with the communities, we have included specific questions that delineate participants’ experiences and perception regarding colonization, residential school enrollment, racism and discrimination. These historical injustices will contextualize the associations between all other predictors and the outcomes of interest.

#### Individual factors

The primary individual factors being considered are the highest level of educational attainment, and lifestyle and behavioural factors, including smoking, physical activity, and alcohol consumption.

#### Covariates

Information was obtained on covariates such as age, sex, body mass index (BMI), waist circumference, and an extensive list of co-morbidities [see Additional file 1].

### Phase 3: Action – address (community-level) and redress (policy-level)

The address-redress phase forms the crux of the FNLHP, where critical community and policy-level interventions informed by baseline assessment results will be implemented to address and redress the issues (exposure-outcome dyads) identified in the logic model (Table 1). With this phase currently being rolled out, a broad implementation structure to address and redress the exposure-outcome dyads has been identified:

1. Housing-asthma: To improve housing conditions by providing tools to reduce dampness and mold, the main focus of the interventions are to provide sustainable tools to improve housekeeping, to devise ways to reduce dampness and mold, and facilitate the adoption of the “Outdoor living room”─ to encourage the elimination of indoor smoking in households with children. Although the ultimate aim of these interventions is to prevent asthma, based on the data obtained by the baseline survey, identification and treatment of asthma, especially in children, is a priority.
2. Smoking-COPD: Interventions to reduce smoking in the community with a specific emphasis on schools.
3. Infections-bronchitis: Interventions to improve hygiene, and increase immunization and flu vaccination rates to ultimately reduce the risk of respiratory illnesses such as bronchitis.
4. Body weight-OSA: To develop upstream interventions which address the obesity pandemic and redress access to health care services specific to the diagnosis and management of OSA.

These interventions are in the preliminary stage of development and will evolve and mature as the FNLHP progresses.

### Interventions in action

#### Environmental study

Housing conditions are known to be associated with respiratory outcomes such as respiratory infections, bronchitis and asthma in First Nations populations. Little is known about the housing conditions responsible for respiratory conditions in Saskatchewan First Nations reserves. Adult participants were asked if they would like to have environmental assessments of their homes. Of the positive responses, 144 homes underwent environmental assessments. Environmental assessments included an interviewer administered housing survey, floor dust collection, and temperature and relative humidity measures. Homes were visited between January and April 2014. Household survey and floor dust data are electronically entered and in the process of being analyzed. Floor and air samples will be assessed gravimetrically and for endotoxin and beta 1–3 glucans.

#### Green light program

The Green Light Program is an evidence-informed and community driven community-level intervention which identifies and celebrates homes that are smoke-free [38]. The objectives of this program are: (i) To celebrate the number of smoke-free homes and increase the number of smoke-free homes by 10% per year; (ii) To engage Elders/mentors/role models/community members in supporting policy related to the misuse of tobacco and community change; (iii) To decrease the rate of tobacco mis-use by 10% per year among all age groups and particularly in youth, pregnant women and seniors; and (iv) To increase cessation strategies by 10% a year among individuals mis- using tobacco. Within the context of this work, tobacco mis-use is defined as non-traditional use of tobacco by First Nations and Métis peoples. Traditional use of tobacco in First Nations and Métis communities is “sacred” and has cultural, medicinal, and spiritual implications which are to be respected, whereas casual or recreational use is mis-use.

#### Addressing and Redressing Obesity-OSA

To address obesity, a two-tiered intervention is being adopted to increase long term physical activity and promote consumption of nutritious food. Toward achieving and sustaining this goal, partnerships with the participating First Nations communities will be used to incorporate not only specific Indigenous knowledge, but also culturally safe and meaningful practices that focus on building community capacity. Similarly, to address OSA, the emphasis will be on utilizing community partnerships to again implement a two-tiered intervention ─ an educational program that not only raises awareness about the long term impact of OSA, but also provides skills in self-administration of a validated questionnaire that highlights the symptoms indicative of OSA risk. This ability to recognize the symptoms of OSA is a critical step in its diagnosis because evidence strongly suggests that an overwhelming majority of the population suffering from OSA do not utilize diagnostic services due to lack of awareness.

To redress obesity, the focus is again on combining Indigenous Knowledge with evidence of food insecurity and the lack of access to places for physical activity in First Nations communities to improve sustainable access. Finally, to redress OSA, it is imperative to appreciate the historical and jurisdictional complexity of healthcare provision to First Nations. Under the Canadian Constitution, healthcare is a provincial responsibility; however, “Registered Indians” are considered under the federal system [39]. As a result, healthcare for First Nations is a complex and complicated endeavour, with services being delivered by the First Nations and the provinces but funded by the federal government, with differing policies and practices, particularly for OSA [40]. Thus, in redressing OSA, a review of healthcare coverage, clinical practices and healthcare utilization is being conducted by interviewing key stakeholders. This multi-dimensional evidence will be combined to identify key gaps in policies and practices that effect First Nations peoples’ ability to utilize medical services for OSA.

### Sampling frame

Based on 2011 Canada Census we used a sampling frame of 321 households and 810 adult residents in Community A, and 259 households and 760 adult residents in Community B. We obtained baseline data from 432 adults (living in 173 households) in Community A and 442 adults (living in 233 households) in Community B (see Table 2)**.**

**Table 2:** **Proposed and observed number of participants in two communities for baseline survey**

	**Community A**		**Community B**	
	**Proposed sample**	**Observed sample (%)**	**Proposed sample**	**Observed sample (%)**
Interviewer-administered questionnaire:				
Households	321^*^	173 (53.9%)	259^*^	233 (89.9%)
Adults	810^**^	432 (53.3%)	760^**^	442 (58.1%)
Clinical measurements:	Consented	Obtained (%)	Consented	Obtained (%)
Lung function	402	326 (81.1%)	379	346 (91.3%)
Allergy test	398	325 (81.6%)	374	345 (92.2%)

^*^Proposed sample of household is based on Band list.

^**^Proposed sample of adults is based on 2011 Canada Census.

### Statistical analysis

Response rates were determined for each of the participating communities. Descriptive results based on pooled data from the participating communities are presented in this manuscript. Statistical analysis was conducted using IBM SPSS Statistics version 21 (IBM Corporation, Armonk, New York). For baseline survey data, descriptive statistics (in terms of frequencies and mean ± standard error) were computed.

## Results

Interviewer-administered surveys were conducted in 2012–2013. Participants included 874 individuals living in 406 households from two reserve communities located in Saskatchewan, Canada. Data were entered and cleaned on an ongoing basis. Final response rates are presented in Table 2. The response rate for household participation was higher in Community B (89.9%) compared to Community A (53.9%). Compared to Community B, Community A is spread over a much wider area resulting in lower response rate due to difficulties in access to the health clinic. Overall, 402 and 398 individuals in Community A and 379 and 374 individuals in Community B consented for lung function and allergy testing respectively. Of these individuals, 326/402 (81.1%) and 325/398 (81.6%) from Community A and 346/379 (91.3%) and 345/374 (92.2%) from Community B completed the lung function and allergy testing respectively.

### Descriptive results based of individual factors, contextual factors and covariates

Four hundred and forty six (51%) females and 428 (49%) males participated in the FNLHP. The mean age of study participants was 35 years (range 17 – 85 years). Approximately 68% of participants were either overweight or obese, 90% were ever smoker (12.1% ex-smoker and 78.0% current smoker) (see Table 3). Frequencies for a number of respiratory health co-morbidities including sleep apnea and Epworth Sleepiness Scale score (a measure of daytime sleepiness) are given in Table 3. Descriptive results for baseline contextual factors are given in Table 4. At the time of the data collection, approximately 70% of the study participants reported their house was either in need of major or minor repairs, 54.7% houses had damage due to dampness, 52.4% houses had mildew/moldy odor, 51% houses had sign of mold or mildew, and 54.2% houses had presence of in-door smoking. The most common source of heating was natural gas.

**Table 3:** **Descriptive results for baseline individual factors and important covariates (N = 874 individuals)**

**(a) COVARIATES**	**N (%)**
**Sex**	
Male	428 (49.0)
Female	446 (51.0)
**Age, in years**	
Mean (SD); range	35.1(14.3); 17-85
≤ 19	110 (12.6)
20-29	285 (32.6)
30-39	174 (19.9)
40-49	135 (15.4)
50-59	116 (13.3)
>=60	54 (6.2)
**Body mass index**	
Mean (SD)	29.1(9.9)
Normal (<25)	252 (32.0)
Overweight (25-30)	238 (30.2)
Obese (>30)	297 (37.7)
**Marital status**	
Married/common law/living together	344 (40.4)
Widowed/divorced/separated single, never married	507 (59.6)
Missing	23 (2.6)
**(b) INDIVIDUAL FACTORS**	N (%)
**Socioeconomic**	
Education	
< Grade 12	430 (49.2)
≥ Grade 12	441 (50.5)
Missing	3 (0.3)
**Lifestyle factors**	
Smoking status	
Current smoker	678 (77.6)
Ex-smoker	109 (12.5)
Never smoker	86 (9.8)
Missing	1 (0.1)
**Health status and co-morbid conditions**	
Perception of health	
Excellent	60 (6.9)
Very good	179 (20.5)
Good	390 (44.6)
Fair	200 (22.9)
Poor	44 (5.0)
Missing	1 (0.1)
**Usually cough on most days for 3 months in a row or more during the year**	
Yes	224 (25.6))
No	646 (74.0)
Missing	4 (0.4)
**Usually bring up phlegm on most days for 3 months in a row or more during the year**	
Yes	264 (30.2)
No	602 (68.9)
Missing	8 (0.9)
**Does your chest ever sound wheezy or whistling, when you have cold**	
Yes	586 (67.1)
No	278 (31.8)
Missing	10 (1.1)
**Does your chest ever sound wheezy or whistling, occasionally apart from colds**	
Yes	317 (36.3)
No	538 (61.5)
Missing	19 (2.2)
**Does your chest ever sound wheezy or whistling, most days or nights**	
Yes	256 (29.3)
No	618 (70.7)
**Shortness of breath**	
Yes	462 (52.9)
No	412 (47.1)
**Respiratory conditions**	
**Doctor diagnosed asthma**	
Yes	150 (17.2)
No	720 (82.4)
Missing	4 (0.4)
**Doctor diagnosed COPD**	
Yes	11 (1.3)
No	826 (94.5)
Missing	37 (4.2)
**Ever had chronic bronchitis**	
Yes	57 (6.5)
No	689 (78.8)
Missing	128 (14.6)
**Ever had emphysema**	
Yes	7 (0.8)
No	734 (84.0)
Missing	133 (15.2)
**Sleep apnea**	
Yes	62 (7.1)
No	683 (78.1)
Missing	129 (14.8)
**Epworth sleepiness scale score**	
Mean (SD); Range	5.4 (4.1); 0-24
Abnormal (>10)	90 (10.3)
Normal (<=10)	738 (84.4)
Missing	46 (5.3)
**Tuberculosis**	
Yes	59 (6.8)
No	685 (78.4)
Missing	130 (14.9)
**Doctor diagnosed diabetes**	
Yes	112 (12.8)
No	728 (83.3)
Missing	34 (3.9)
**Doctor diagnosed heart problems**	
Yes	86 (9.8)
No	746 (85.4)
Missing	42 (4.8)
**Doctor diagnosed stroke**	
Yes	12 (1.4)
No	836 (95.7)
Missing	26 (3.0)
**Doctor diagnosed depression**	
Yes	161 (18.4)
No	668 (76.4)
Missing	45 (5.1)
**Doctor diagnosed kidney problem**	
Yes	65 (7.4)
No	773 (88.4)
Missing	36 (4.1)
**Doctor diagnosed leg ulcers or amputations**	
Yes	18 (2.1)
No	831 (95.1)
Missing	25 (2.9)
**Doctor diagnosed eyesight problems**	
Yes	77 (8.8)
No	771 (88.2)
Missing	26 (3.0)
**Doctor diagnosed cancer**	
Yes	29 (3.3)
No	822 (94.1)
Missing	23 (2.6)

**Table 4:** **Descriptive results for baseline contextual factors (N = 406 households)**

**CONTEXTUAL FACTORS**	**N (%)**
**Family/household structure**	
**Number of people**	
Mean ± SE	5.02 ± 0.081
Range	1-14
**Number of bedrooms**	
Mean ± SE	5.37 ± 0.05
Range	1-10
**Band-owned home**	
Yes	359 (88.4)
No	36 (8.9)
Do not know	11 (2.7)
**Housing conditions**	
**House in need of repairs**	
Yes, major repairs	155 (38.2)
Yes, minor repairs	119 (29.3)
No, only regular maintenance is required	119 (29.3)
Missing	13 (3.2)
**Water or dampness in your house from broken pipes, leaks, septic tank, heavy rain, or floods**	
Yes	229 (56.4)
No	151 (37.2)
Do not know	23 (5.7)
Missing	3 (0.7)
**House have any damage caused by dampness**	
Yes	207 (51.0)
No	174 (42.9)
Do not know	22 (5.4)
Missing	3 (0.7)
**House have a mildew/moldy odor or musty smell**	
Yes	199 (49.0)
No	178 (43.8)
Do not know	25 (6.2)
Missing	4 (1.0)
**Signs of mold or mildew in any living areas of house**	
Yes	190 (46.8)
No	178 (43.8)
Do not know	34 (8.4)
Missing	4 (1.0)
**Any people who live in your house smoke in the house**	
Yes	216 (53.2)
No	189 (46.5)
Missing	1 (0.3)

## Discussion

Aboriginal Peoples’ respiratory health inequalities pose challenges that are both complex and unique. Such challenges may be attributed to inequities such as Aboriginal Peoples’ socioeconomic disadvantages [18], which are driven by historical trauma related to colonization [15] and residential school enrollment [16,17]. More importantly, the intergenerational link between inequities, inequalities and health outcomes magnifies the challenges faced by Aboriginal communities.

Moreover, the faster growth of the Aboriginal population highlights the widening health inequalities which will be economically unsustainable with current trends in Canadian population growth. Furthermore, with poor household conditions increasing the risk of chronic respiratory illnesses in children as well [11], the disproportionately larger younger Aboriginal population increases the risk of continued intergenerational transmission of health inequalities [17,19]. The respiratory health risks faced by Aboriginal peoples are likely further accentuated in rural Aboriginal populations due to their geographic and economic isolation.

Thus, in taking a step towards addressing and redressing respiratory health inequalities in rural Aboriginal populations, the FNLHP has been conceived as a community-based participatory research initiative in two on-reserve First Nations communities in Saskatchewan. As FNLHP involves the participating communities at each phase of its implementation, the objective of this manuscript was to describe the rationale and development of the community-based participatory research methodology. This methodology lays the foundation for the intervention phases of FNLHP ─ address (community-level) and redress (policy-level).

Acknowledging the complexity of targeting inequalities in these vulnerable populations, the methodology of this project is informed by the logic model (Table 1) which not only determines the phases of the project, but also enumerates how the selected exposure-outcome dyads will be addressed, redressed and reassessed. Selection of these dyads in consultation with the communities was a critical first step of the project. First, it provided direction to the entire project in terms of targeting the issues that were important to the community. More importantly, however, through the “vision and relationships” process, the selection of respiratory issues enabled vital trust building between the researchers and the communities. This trust will be invaluable for the successful completion of the later stages of the project.

Baseline assessment which followed problem identification was again enabled by community participation. The questionnaire was developed and field tested with direction from community leaders, which ensured its smooth deployment during the final data collection. The trust gained through these processes encouraged community members to participate in clinical testing. The survey questionnaires were developed taking into account PHF’s framework and thus captured a wide range of contextual and individual determinants that could influence respiratory health. These determinants also included the impact of colonization and racism that ultimately drive the socio-economic challenges of First Nations peoples. Both the questionnaires and the clinical tests will generate evidence that will drive the address and redress phases of the project.

One of the limitations of our study is the low response rate in Community A compared to Community B. The explanation for this low response rate could be due to transportation problem (because Community A is quite spread out) to the health clinic where baseline survey was conducted, lung function measures were obtained, and allergy test were done. Another limitation is that we hired local people to conduct interviews and due to closeness among community members could lead to information bias.

The interventions in action highlight the multi-dimensional nature of this project. The concurrent implementation of community and policy-level interventions targeting each identified exposure-outcome dyad is a complex process. The need for individualized research expertise is obvious to successfully carry out these interventions, however, without the participation and uptake of the interventions by the community, it would be unrealistic to achieve the goals of this project.

## Conclusions

The information from this project will assist in addressing and redressing many of the issues involved including the provision of adequate housing, health lifestyle practices, and in planning for health service delivery. Even though the immediate goals are to address and redress respiratory health inequalities, the ultimate aim of this project is to build sustainable community capacity by deriving and integrating Indigenous Knowledge with western research methodologies. Thus, this community-based participatory methodology will not only increase the chances of improved long term respiratory health outcomes, but it will also facilitate future interventions targeting other health issues of importance to First Nations communities.

## Additional file

Additional file 1:
**Appendix A.**

## References

[1] University of British Columbia. First Nations Studies Program. 2009. Terminology – Available from http://indigenousfoundations.arts.ubc.ca/home/identity/terminology.html?type=123&filename=Terminology.pdf] Accessed on February 13, 2015 - accessed on February 13, 2015.

[2] Health Canada. 2009. A statistical profile on health of First Nations in Canada: Determinants of Health 1999–2003. Health Canada: Ottawa, ON, Canada. http://www.hc-sc.gc.ca/fniah-spnia/alt_formats/fnihb-dgspni/pdf/pubs/aborig-autoch/2009-stats-profil-eng.pdf. Accessed on Feb 5, 2015.

[3] Statistics Canada. 2008. Aboriginal People in Canada in 2006: Inuit, Metis and First Nations, 2006 Census. Statistics Canada: Ottawa, ON, Canada. http://www12.statcan.ca/census-recensement/2006/as-sa/97-558/pdf/97-558-XIE2006001.pdf. Accessed on September 20, 2014.

[4] Barsh R (1994). Canada’s Aboriginal people: social integration or disintegration?. Can J Nativ Stud.

[5] MacMillan HL, MacMillan AB, Offord DR, Dingle JL (1996). Aboriginal health. CMAJ.

[6] Janz T, Seto J, Turner A. Aboriginal Peoples Survey, 2006: An overview of the Health of the Metis Population, Statistics Canada: Ottawa, ON, Canada; 2009. http://www.statcan.gc.ca/pub/89-637-x/89-637-x2009004-eng.pdf. Accessed on Sep 10, 2014.

[7] Melia RJW, Chinn S, Rona R (1988). Respiratory illness and home environment of ethnic groups. BMJ.

[8] Crighton E, Wilson K, Senecal S (2010). The relationship between socio-economic and geographical factors and asthma among Canada’s Aboriginal populations. Int J Circumpolar Health.

[9] Reading CL, Wien E. Health Inequalities and Social Determinants of Aboriginal Peoples’ Health. National Collaborating Centre for Aboriginal Health: Prince George, BC, Canada, 2009. http://www.nccah-ccnsa.ca/docs/social%20determinates/nccah-loppie-wien_report.pdf. Accessed on August 10, 2014.

[10] King M, Smith A, Gracey M (2009). Indigenous health part 2: the underlying causes of the health gap. Lancet.

[11] Reading J (1999). An examination of residential schools and elder health. Chapter 2. First Nations and Inuit regional health survey. National Report.

[12] Smith D, Varcoe C, Edwards N (2005). Turning around the intergenerational impact of residential schools on Aboriginal people: implications for health policy and practices. CJNR.

[13] First Nations Centre. 2005. First Nations Information Governance Committee. First Nations Regional Longitudinal Health Survey (RHS) 2002/03. Results for adult, youth and children living in First Nations Communities. First Nations Centre: Ottawa, ON, Canada. http://fnigc.ca/sites/default/files/ENpdf/RHS_2002/rhs2002-03-technical_report.pdf. Accessed on July 23, 2014.

[14] Statistics Canada. 2007. Saskatchewan (Code47) *(table).* Aboriginal Population Profile*.* 2006 Census. Catalogue no. 92-594-XWE. Statistics Canada: Ottawa, ON, Canada. Released January 15, 2008. http://www12.statcan.ca/census-recensement/2006/dp-pd/prof/92-594/index.cfm?Lang=E Accessed on May 4, 2014.

[15] Lawrence R, Martin D (2001). Moulds, moisture and microbial contamination of First Nations housing in British Columbia, Canada. Int J Circumpolar Health.

[16] Rizzo MC, Naspitz CK, Fernandez-Calda E, LeeKey RF, Mimica I, Sole D (1997). Endotoxin exposure and symptoms in asthmatic children. Pediatr Allergy Immunol.

[17] Park JH, Gold R, Spiegelman DL, Burge HA, Milton D (2001). House dust endotoxin and wheeze in the first year of life. Am J Respir Crit Care Med.

[18] Michel O, Kips J, Dutchateau J, Vertongen F, Robert L, Collet H (1996). Severity of asthma is related to endotoxin in house dust. Am J Respir Crit Care Med.

[19] Brunekreef B, Dockery DW, Speizer FE, Ware JH, Spengler JD, Ferris BG (1989). Home dampness and respiratory morbidity in children. An Rev Respir Dis.

[20] Sin DD, Wells H, Svenson LW, Man SFP (2002). Asthma and COPD among Aboriginals in Alberta, Canada. Chest.

[21] Dales RE, Burnett R, Zwanenburg H (1991). Adverse health effects among adults exposed to home dampness and molds. Am Rev Respir Dis.

[22] Clark M, Riben P, Nowgesic E (2002). The association of housing density, isolation and tuberculosis in Canadian First Nations communities. Int J Epidemiol.

[23] Kovesi T, Gilbert N, Stocco C, Fugler D, Dales R, Guay M (2007). Indoor air quality and the risk of lower respiratory tract infections in young Canadian Inuit children. CMAJ.

[24] Statistics Canada. 2014. Aboriginal Peoples in Canada: First Nations People, Metis, and Inuit. National Household Survey 2011. Statistics Canada: Ottawa, ON, Canada. http://www12.statcan.gc.ca/nhs-enm/2011/as-sa/99-011-x/99-011-x2011001-eng.pdf. Accessed on June 10, 2014.

[25] Anderson A. Aboriginal Populations Trends. The Encyclopedia of Saskatchewan. http://esask.uregina.ca/entry/aboriginal_population_trends.html. Accessed on May 5, 2014.

[26] Ministerial Advisory Committee on Rural Health. 2002. Rural Health in Rural Hands: Strategic Directions for Rural, Remote, Northern and Aboriginal Communities. Health Canada: Ottawa, ON, Canada. http://www.ruralontarioinstitute.ca/file.aspx?id=29b5ba0b-c6ce-489f-bb07-2febfb576daa. Accessed on September 12, 2014.

[27] Kirby M, LeBreton M. The Health of Canadians: The Federal Role. Vol 6, Recommendations for reform. The Standing Senate Committee on Social Affairs, Science and Technology. Parliament of Canada: Ottawa, ON, Canada; 2002. http://www.parl.gc.ca/Content/SEN/Committee/372/soci/rep/repoct02vol6-e.pdf. Accessed on June 10, 2014.

[28] Romanow RJ. Building on values: The future of health care in Canada. Commission on the Future of Health Care in Canada. National Library of Canada: Ottawa, ON, Canada; 2002. http://www.cbc.ca/healthcare/final_report.pdf. Accessed on Dec 15, 2014.

[29] Canadian Institute for Health Information. 2006. How healthy are rural Canadians? An assessment of their health status and health determinants. A component of the initiative **“**Canada’s rural communities: Understanding rural health and its determinants**”**. Canadian Institute for Health Information: Ottawa, ON, Canada. https://secure.cihi.ca/free_products/rural_canadians_2006_report_e.pdf. Accessed on November 6, 2014.

[30] Health Canada. 1994. Strategies for population health: investing in the health of Canadians. Minister of Supply and Services Canada. Health Canada: Ottawa, ON, Canada. http://publications.gc.ca/collections/Collection/H88-3-30-2001/pdfs/other/strat_e.pdf. Accessed on Sep 4, 2014.

[31] Pickett W, Day L, Brison RJ, Marlenga BL, Pahwa P, Koehncke N (2008). The Saskatchewan farm injury Cohort: rationale and methodology. Public Health Rep.

[32] Pahwa P, Karunanayake C, Hagel L, Janzen B, Pickett W, Rennie D, et. al. The Saskatchewan rural health study: an application of a population health framework to understand respiratory health outcomes. BMC Research Notes 2012, 5:400 http://www.biomedcentral.com/1756-0500/5/400 10.1186/1756-0500-5-400 PMC3438108 22852584

[33] Senthilselvan A, Rennie DC, Chénard L, Burch LH, Babiuk L, Schwartz DA (2008). Association of polymorphisms of toll-like receptor 4 with a reduced prevalence of hay fever and atopy. Ann Allergy Asthma Immunol.

[34] Senthilselvan A, Dosman JA, Pahwa P (1991). Respiratory symptoms and alterations in pulmonary tests in poultry producers. Am Rev Respir Dis.

[35] Chénard L, Senthilselvan A, Grover VK, Kirychuk SP, Lawson JA, Hurst TS (2007). Lung function and farm size predict healthy worker effect in swine farmers. Chest.

[36] Miller MR, Hankinson J, Brusasco V, Burgos F, Casaburi R, Coates A (2005). Standardization of Spirometry. Eur Respir J.

[37] Joint Task Force on Practice Parameters, American Academy of Allergy, Asthma and Immunology, American College of Allergy, Asthma and Immunology, Joint Council of Allergy, Asthma and Immunology (2007). Allergen immunotherapy: a practice parameter second update. J Allergy Clin Immuno.

[38] Ramsden VR, McKay S, Bighead S, Boucher G, Bourassa C, Butt P (2013). Hypothesis - participatory health research: celebrating smoke free homes. Can Fam Physician.

[39] Government of Canada. 2014. **“**The Indian Act**”**. Indian Act. Department of Justice Canada: Ottawa, ON, Canada. http://laws-lois.justice.gc.ca/eng/acts/i-5/20130401/P1TT3xt3.html. Accessed on March 16, 2014

[40] Health Canada. 2009. Provider Guide for Medical Supplies and Equipment (MS&E). Benefits: Non-Insured Health Benefits. Health Canada: Ottawa, ON, Canada. http://www.hc-sc.gc.ca/fniah-spnia/alt_formats/fnihb-dgspni/pdf/pubs/medequip/2009-prov-fourn-guide-eng.pdf. Accessed March 7, 2014.

