# Supplementary material for: A community-based participatory research methodology to address, redress, and reassess disparities in respiratory health among First Nations
Source: BMC Res Notes. 2015 May 16;8:199. doi: 10.1186/s13104-015-1137-5 (PMC4440281; doi:10.1186/s13104-015-1137-5)
Supplement: Additional file 1: — Appendix A. [file 13104_2015_1137_MOESM1_ESM.doc]

**APPENDIX A**

***Perceived financial strain was based on the following question:***

At the end of the month, how much money do you have left over?

*Please check only one.*

- - - Some money
    - Just enough money
    - Not enough money

***Social support was based on the following question:***

***-*** Have you ever experienced discrimination or racism, been prevented from doing something, or been hassled or made to feel inferior (badly) in any of the following situations because of your race, cultural group or color.

At school? □ Yes □ No

Getting hired or getting a job? □ Yes □ No

At work? □ Yes □ No

Getting housing? □ Yes □ No

Getting medical care? □ Yes □ No

Getting medicine at pharmacies? □ Yes □ No

Getting service in a store or restaurant? □ Yes □ No

Getting credit, bank loans or a mortgage? □ Yes □ No

On the street or in a public setting? □ Yes □ No

From the police or in the courts? □ Yes □ No

Getting a cheque cashed? □ Yes □ No

- What are the main strengths of your community (village)?

#### Check all that apply.

- - - Family values
    - Awareness of First Nations culture
    - Social connections (community working together)
    - Traditional ceremonial activities (e.g. powwow)
    - Low rates of suicide/crime/drug abuse
    - Good leisure/recreation facilities, elders
    - Use of First Nations language
    - Education and training opportunities
    - Natural environment
    - Strong economy
    - Strong leadership
    - Other:

**Colonization was based on the following question:**

For the purpose of the survey, the term ‘residential schools’ refers to the residential school systems attended by Aboriginal students. This includes residential schools run by religious orders, industrial schools, boarding schools, student residences, hostels and billets. The last residential school shut down in 1996.

1. Did you attend a residential school?
   - Yes
   - No
   - Don’t know
2. Did either of your parents or grandparents attend a residential school?
   - Yes
   - No
   - Don’t know

**List of co-morbidities:**

***-*** Has a doctor or primary care giver ever said you have?

|  | **Yes** | **No** | **Do not Know** |  |
| --- | --- | --- | --- | --- |
| Diabetes | □ | □ | □ |  |
| Heart problems | □ | □ | □ | If YES, did you have a heart attack?   - Yes □ No |
| Stroke | □ | □ | □ |  |
| Depression | □ | □ | □ |  |
| Kidney problems | □ | □ | □ |  |
| Leg ulcers or amputations | □ | □ | □ |  |
| Severe eyesight problems (not including a need for glasses) | □ | □ | □ |  |
| Cancer | □ | □ | □ | If YES, please specify: |
| Other | □ | □ | □ | If YES, please specify: |

- During the past twelve months, were you seen by a doctor or other primary care giver for:

|  | **Yes** | **No** | **Do not know know** |
| --- | --- | --- | --- |
| heartburn? | □ | □ | □ |
| an ear infection? | □ | □ | □ |
| an injury? | □ | □ | □ |
